# Sleep Medicine—What's in a Name?

**DOI:** 10.1111/jsr.70150

**Published:** 2025-07-19

**Authors:** Dirk A. Pevernagie, Erna Sif Arnardottir, Oliviero Bruni, Sarah Hartley, Gert Jan Lammers, Tiina Paunio, Dieter Riemann, Renata L. Riha

**Affiliations:** ^1^ Department of Internal Medicine and Paediatrics Faculty of Medicine and Health Sciences, Ghent University Gent Belgium; ^2^ Department of Respiratory Medicine Roeselare Belgium; ^3^ Reykjavik University Sleep Institute School of Technology, Reykjavik University Reykjavik Iceland; ^4^ Developmental and Social Psychology, Paediatric Sleep Clinic Sapienza University Rome Italy; ^5^ Sleep Clinic APHP Hôpital Raymond Poincaré Paris France; ^6^ Department of Neurology Leiden University Medical Centre Leiden the Netherlands; ^7^ Sleep Wake Centre Stichting Epilepsie Instellingen Nederland (SEIN) Heemstede the Netherlands; ^8^ Department of Psychiatry and SleepWell Research Program Faculty of Medicine, University of Helsinki and Helsinki University Hospital Helsinki Finland; ^9^ Department of Psychiatry and Psychotherapy Medical Centre, Faculty of Medicine, University of Freiburg Breisgau Germany; ^10^ Department of Sleep Medicine University of Edinburgh, Royal Infirmary Edinburgh Edinburgh UK

**Keywords:** classification, future, history, research, sleep medicine, uncertainty

## Abstract

Sleep medicine has matured into a recognised medical discipline, characterised by defined diagnostic concepts, evidence‐based treatments, and significant progress in understanding sleep physiology and disorders. Sleep and its disturbances impact virtually every aspect of health and well‐being. The major categories of sleep disorders include insomnia, neurological and psychiatric sleep disorders, sleep‐disordered breathing, and paediatric sleep disorders. Breakthroughs in biomedical research have deepened clinical expertise across each of these domains. Although sleep medicine has historically developed from various specialties, the current approach emphasises interdisciplinary collaboration. Today, diagnostic and therapeutic pathways are well established, supported by professional standards outlined in nosological classifications, clinical guidelines, and structured frameworks of competencies and skills. Despite the universal importance of sleep and the high prevalence of sleep disorders, the field continues to face systemic challenges—most notably limited access to care, inadequate funding for clinical services, and insufficient investment in research. The central challenge is to balance the integration of new opportunities with the resolution of persistent uncertainties. However, advances in technology and the emergence of precision medicine offer promising prospects for progress. Sleep medicine stands at a crossroads. Its future will depend on rearticulating its mission and vision, addressing structural shortcomings, embracing innovation, and affirming its essential role in promoting public health.

**Abbreviations**
3P model: predisposing, precipitating, and perpetuating factors of insomnia
AASM: American Academy of Sleep Medicine
AHI: apnoea‐hypopnoea index
AI: artificial intelligence
CBT‐I: cognitive behavioural therapy for insomnia
dCBT‐I: digital cognitive behavioural therapy for insomnia
DORAs: dual orexin receptor antagonists
CPAP: continuous positive airway pressure
DSM: diagnostic and statistical manual of mental disorders
EDS: excessive daytime sleepiness
EEG: electro‐encephalography
EMA: European Medicines Agency
EMG: electro‐myography
EOG: electro‐oculography
EPSC: European Paediatric Sleep Club
ESRS: European Sleep Research Society
FDA: Food and Drug Administration (USA)
ICSD: international classification of sleep disorders
IH: idiopathic hypersomnia
MRA: mandibular advancement device
MSLT: multiple sleep latency test
NREM sleep: non‐rapid eye movement sleep
OSA: obstructive sleep apnoea
PSG: polysomnography
RBD: REM sleep behaviour disorder
RCTs: randomised controlled trials
REM sleep: rapid eye movement sleep
RLS: restless legs syndrome
RSI: repetitive strain injury
SDB: sleep‐disordered breathing
SIDS: sudden infant death syndrome

*All scientific work is incomplete. All scientific work is liable to be upset or modified by advancing knowledge. That does not confer upon us a freedom to ignore the knowledge we already have, or to postpone the action that it appears to demand at a given time*.

—Sir Austin Bradford Hill 1965

## 1 Introduction

### 1.1 Historical notes

Over the past few decades, the field of diagnosing and treating sleep disorders has evolved into a distinct and autonomous discipline within healthcare. Sleep medicine has developed along a trajectory similarly to other major medical specialties, progressing through stages of scientific discovery and clinical refinement. The formal recognition of any medical specialty typically follows the emergence of relevant new concepts in medical science, and sleep medicine is no exception. The milestones of fundamental research in sleep neurobiology and molecular mechanisms of circadian rhythmicity have been extensively described in other papers (Huang 2018; Schulz 2022), and are beyond the scope of this review.

**FIGURE 1:**
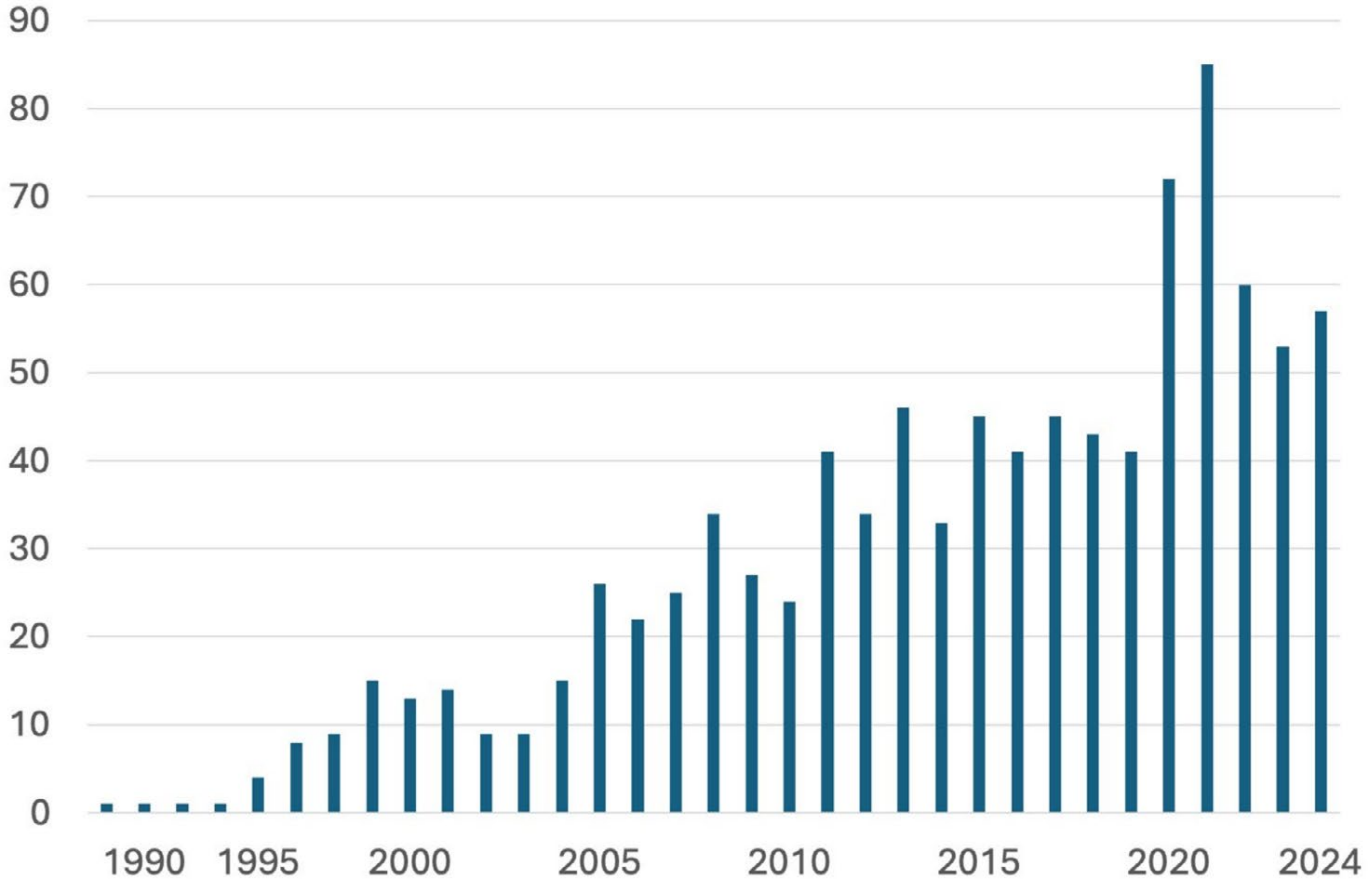
Frequency of the term ‘Sleep Medicine’ in the title of papers indexed in PubMed.

Sustained basic and clinical research in sleep science has generated a wealth of knowledge, firmly establishing sleep medicine as a medical discipline in its own right (Shepard et al. 2005). Initially, sleep disorders were poorly understood and addressed with generalised treatments and broad recommendations. Today, specific diagnostic and therapeutic options are available for clearly defined conditions. Sleep medicine now boasts its own taxonomy (AASM 2023), accompanied by standardised diagnostic criteria and targeted treatment protocols, consolidating its place in modern healthcare. Moreover, the name ‘sleep medicine’ has increasingly been mentioned in the title of medical journals since its first appearance in 1988 (Lavie 1988) (Figure 1). The term ‘paediatric sleep medicine’ first appeared in a paper title at the turn of the century (Owens 2001), and has since been published under this designation at a rate of 1–4 items per year.

Groundbreaking biomedical discoveries across various disciplines have contributed to our understanding of sleep mechanisms and related pathologies. Table 1 presents a selected anthology of these accomplishments. In this paper, we aim to provide a concise overview of significant advances in insomnia, neurology, psychiatry, respiratory medicine, and paediatric sleep medicine below.

**TABLE 1:** Milestones in sleep research and the development of sleep medicine.

Year	Authors	Revelant fact
1880	Gélineau J.	Introduction of the name ‘narcolepsy’
1934	Daniels L.	First comprehensive description of symptoms in narcolepsy
1939	Kleitman N.	Sleep and wakefulness: The first comprehensive scientific publication on the physiology of sleep and wakefulness
1950	Ekbom K.	The first large series of patients with restless legs syndrome
1953	Aserinsky E. and Kleitman N.	Description of REM sleep in infants
1956	Burwell C. et al.	Pickwickian syndrome
1957	Yoss R. and Daly D.	Systematic description of narcolepsy in children
1957	Dement W. and Kleitman N.	Description of cyclically alternating NREM and REM cycles
1963	Jones D. and Jones E.	Overview of drugs for insomnia
1965	Jouvet M. and Delorme F.	Induction of behavioural acts during paradoxical sleep in cats with lesions in the pontine tegmentum
1968	Rechtschaffen A. and Kales A.	A manual of standardised terminology, techniques and scoring system for sleep stages of human subjects
1971	Konopka R. and Benzer S.	Clock genes identified in *Drosophila melanogaster*
1976	Guilleminault C., Dement W. et al.	Description of the sleep apnoea syndromes
1979	Association of Sleep Disorders Centers	First classification of sleep disorders
1981	Sullivan C. et al.	CPAP therapy as an effective treatment of obstructive sleep apnoea
1983	Honda Y. et al.	Discrimination of narcolepsy by using genetic markers and HLA
1985	Terzano M. et al.	Description of cyclic alternating pattern
1986	Schenck C. et al.	Discovery of REM sleep behaviour disorder
1987	Spielman A et al.	The 3P model of insomnia and description of sleep restriction as a new therapy for insomnia
1989	Kryger M. et al.	Principles and Practice of Sleep Medicine, first edition
1990	American Sleep Disorders Association	International Classification of Sleep Disorders; first edition
1995	Ferber R. and Kryger M.	Principles and Practice of Sleep Medicine in the Child, first edition
1998	de Lecea L. et al.	Discovery of hypocretin neurotransmission
1998	Sakurai T. et al.	Discovery of orexin neurotransmission
1999	Morin C. et al.	First RCT on cognitive behavioural therapy of insomnia
2000	Nishino S. et al.	Hypocretin (orexin) deficiency in human narcolepsy
2009	Ritterband L. et al.	First RCT on digital cognitive behavioural therapy of insomnia
2012	European Sleep Research Society	First European Somnology examination
2013	Eckert D. et al.	Description of the endotypic features of obstructive sleep apnoea
2014	Penzel T. et al.	Catalogue of knowledge and skills in sleep medicine
2014	European Sleep Research Society (Bassetti C., Dogas Z. and Peigneux P.)	European Sleep Medicine Textbook, first edition
2016	Qaseem A. et al.	The American College of Physicians denotes in its guidelines for the first time ever CBT‐I as first line treatment for insomnia
2017	Hall J., Rosbach M., and Young M.	Nobel Prize for the discovery of molecular mechanisms controlling the circadian rhythm
2017	Riemann D. et al.	European guideline for the diagnosis and treatment of insomnia
2021	Arnardottir E. et al.	Sleep revolution: recipient of major research grant from the EU

[Correction added on 26 July 2025, after first online publication: The Revelant Fact of Terzano M. et al. has been updated in this version.]

### 1.2 Insomnia Disorder

Acute, transient insomnia affects most people at least once in their lifetime, while chronic insomnia impacts up to 10% of the general population. Given its high prevalence, it is arguably the most common sleep disorder. Chronic insomnia is coupled to a high disease burden and a distinctly reduced quality of life (Morin et al. 2015).

Historical accounts of insomnia date back to ancient times, with various causes and treatments described in both the medical and lay literature. In pre‐modern times, a sinful life was often considered to be a major cause of sleeplessness. Remedies ranged from substances like opiates and alcohol to lifestyle modifications—early forms of what we now recognise as sleep hygiene (Ahlheim 2018).

In modern medical classification, insomnia is defined by subjective symptoms, including at least one sleep complaint—such as difficulty falling asleep, maintaining sleep, or waking too early—accompanied by significant daytime impairment (e.g., fatigue, poor concentration, mood disturbances, and heightened anxiety) (Riemann et al. 2023).

Until recently, insomnia was primarily regarded as a symptom secondary to other medical or psychiatric conditions, with treatment focused on addressing the potential underlying disorder. However, with the publication of the fifth edition of the Diagnostic and Statistical Manual of Mental Disorders (DSM) (APA 2013) and the third edition of the International Classification of Sleep Disorders (ICSD) published by the American Academy of Sleep Medicine (AASM) (AASM 2014), the term ‘insomnia disorder’ emerged, acknowledging growing evidence that insomnia is an independent condition warranting its own diagnostic and therapeutic approach.

Research has since confirmed that insomnia is a significant, independent risk factor for cardiometabolic diseases and psychiatric disorders. Studies have demonstrated that effective insomnia treatment is associated with a reduced incidence of depression and relapse prevention (Furukawa et al. 2024; Hertenstein et al. 2019).

Psychological models, such as the 3P model (predisposing, precipitating, and perpetuating factors) and cognitive models, have long formed the foundation of insomnia research (Spielman et al. 1987). More recently, the concept of 24‐h hyperarousal—a state of mental overactivity—has been implicated in its pathophysiology (Dressle and Riemann 2023). While traditionally identified through psychological assessments, emerging evidence suggests underlying neurobiological mechanisms (Palagini et al. 2023; Riemann et al. 2025).

Insomnia treatment encompasses both pharmacological and non‐pharmacological strategies (Riemann et al. 2023). Pharmacological treatments have evolved from classical benzodiazepines to benzodiazepine receptor agonists, melatonin, sedating antidepressants, and newer orexin receptor antagonists. Non‐pharmacological treatments, particularly cognitive behavioural therapy for insomnia (CBT‐I), focus on techniques such as stimulus control, sleep restriction, and cognitive restructuring. Sleep hygiene practices, including reducing caffeine, nicotine, and alcohol intake, also play a role. Current insomnia treatment guidelines strongly advocate CBT‐I as the first‐line therapy due to its efficacy and the potential risks associated with medication, such as dependence. However, due to a shortage of trained CBT‐I therapists in many regions, digital CBT‐I (dCBT‐I) has emerged as a promising alternative, expanding access to evidence‐based insomnia care.

### 1.3 Neurological Sleep Disorders

Neurological sleep disorders encompass a wide range of conditions associated with diseases of the central nervous system. Among these, narcolepsy, restless legs syndrome (RLS), and rapid eye movement (REM) sleep behaviour disorder (RBD) stand out as prominent and well‐characterised syndromes. Each of these is briefly described below.

#### 1.3.1 Narcolepsy

Narcolepsy is a prototypical neurological sleep disorder. The study of this condition has significantly advanced our understanding of sleep regulation, the underlying neural structures, neurotransmitter systems, and the intricate relationships between sleep, metabolism, and other basic functions (Saper et al. 2001).

The condition was first described by Carl Friedrich Otto Westphal in 1877 (Westphal 1877), and the term ‘narcolepsy’ was coined by Jean‐Baptiste Gelineau in 1880 (Gélineau 1880). From the first descriptions, debate persisted—well into the 1960s—about whether narcolepsy with cataplexy constituted a distinct entity and whether it was neurological versus psychiatric or nonorganic in origin.

Lemur Daniels published a seminal paper on narcolepsy in 1934, already noting disturbed nocturnal sleep and weight gain as part of the clinical presentation, besides the typical excessive daytime sleepiness (EDS), hypnagogic hallucinations, and sleep paralysis (Daniels 1934). Unfortunately, later work at the Mayo Clinic promoted a tetrad of narcolepsy symptoms, whereby the first two symptoms were largely overlooked (Yoss and Daly 1957).

In 1960, it was discovered that patients with narcolepsy, unlike healthy people, typically enter REM sleep almost immediately upon falling asleep during the day. Initially, this was seen as a ‘proof’ of Freud's psychodynamic theory of dream fulfilment, whereby people experiencing real‐life issues satisfy their unfulfilled desires in dreaming content (Vogel 1960). However, this interpretation shifted dramatically when William Dement and coworkers bred a colony of narcoleptic dogs at Stanford, showing that narcolepsy‐like symptoms observed in humans could also occur in animals (Peyron et al. 2000). This effectively ended the debate over whether or not narcolepsy was an organic disorder.

The major breakthrough came when researchers from the Stanford/Leiden group discovered that narcolepsy with cataplexy in humans is caused by a deficiency of hypocretin, also known as orexin (Nishino et al. 2000). This seminal discovery was made possible by prior research in Stanford that identified a mutation in the hypocretin receptor as the cause of an inherited form of narcolepsy in Doberman Pinschers (Lin et al. 1999).

These findings have paved the way for the development of near‐causal treatments with hypocretin agonists. The first phase 3 clinical trial is currently underway, following quite convincing results from phase 2 studies (Dauvilliers 2025). Yet, the long‐term efficacy and safety of these treatments remain to be proven in future studies.

The underlying cause of hypocretin deficiency in humans is still unclear, although substantial evidence suggests that an autoimmune process may be involved (Latorre et al. 2018). However, this hypothesis has yet to be definitively proven.

#### 1.3.2 Restless Legs Syndrome (RLS)

Sir Thomas Willis provided the first known medical description of RLS (Willis 1672, 1685). He clearly described the characteristic limb movements and associated sleep disruption in those affected. He noted patients as being ‘…no more able to sleep, than if they were in a place of the greatest torture’. For centuries, RLS was considered a functional or psychiatric disorder. It wasn't until 1888 that Dr. G. M. Beard proposed a spinal origin for the motor restlessness (Beard 1888).

In 1945, Swedish neurologist Karl‐Axel Ekbom provided a comprehensive clinical account of the condition in his doctoral thesis, coining the term ‘restless legs’ (Ekbom 1950). His publication was crucial in establishing RLS as a distinct clinical entity. In 1995, the formation of the International Restless Legs Syndrome Study Group further elaborated on the definition of its clinical criteria (Walters 1995).

Although RLS frequently runs in families and was initially believed to follow a classical monogenic inheritance pattern, genetic studies in affected families have not yet identified definitive causative genes (Winkelmann et al. 2017). While several risk loci and genetic variants have been demonstrated according to large genome‐wide association studies (Schormair 2025), the understanding of the genetic predisposition remains incomplete.

It is likely that RLS is a complex sensorimotor disorder with the involvement of cortical, subcortical, spinal cord, and peripheral nerve generators in a network disorder, leading to enhanced excitability and/or decreased inhibition (Lanza et al. 2017). The presence of low ferritin concentrations in the cerebrospinal fluid and reduced iron stores in the brains of patients with restless legs syndrome suggests that iron transport into neurons is impaired (Li et al. 2016). Nonetheless, full insight into pathophysiological mechanisms has yet to be established.

In the presence of iron deficiency, the syndrome may respond rapidly to iron treatment. In patients without iron deficiency, treatment was revolutionised by the use of dopamine agonists, but over time the high incidence of adverse effects has encouraged the use of other treatments such as alpha‐2‐delta ligands and opiates (Trenkwalder et al. 2024; Winkelman et al. 2025).

#### 1.3.3 REM Sleep Behaviour Disorder (RBD)

RBD was first described in humans in 1986 by Carlos Schenck and collaborators (Schenck et al. 1986). Prior to this, dream enactment behaviour—referred to as ‘oneiric activity’—had been observed in animal studies. More than 20 years earlier, Michel Jouvet and his team showed that cats with selective bilateral lesions in the pontine tegmentum displayed stereotypical behaviours during REM sleep, suggesting the role of this brain region in regulating REM atonia (Luppi 2018).

By 1996, follow‐up studies revealed that 11 of the 29 patients in Schenck's original cohort had developed parkinsonism (Schenck et al. 1996). Subsequent reports on other longitudinal surveys confirmed that RBD may be an early clinical manifestation of α‐synucleinopathy, that is, neurodegenerative disease caused by abnormal accumulation of aggregates of alpha‐synuclein protein in neurons, nerve fibres, or glial cells (McCann et al. 2014). There are three main types of synucleinopathy: Parkinson's disease, dementia with Lewy bodies, and multiple system atrophy. It is assumed that tegmental pontine neurons that regulate REM sleep atonia are affected in RBD. From the outset, RBD has been recognised as an organic neurological disorder and is now considered a relevant early marker of these neurodegenerative diseases (Iranzo et al. 2013).

Pharmacological treatment aims at controlling symptoms; no causal remedy is as yet available (During et al. 2025).

### 1.4 Sleep and Psychiatric Disorders

Following the discovery of REM sleep in 1953, a number of pioneering researchers began exploring sleep patterns in individuals with psychiatric disorders—most notably affective disorders and schizophrenia. Although early studies hoped to uncover specific sleep abnormalities tied to particular psychiatric conditions, these expectations were largely unmet. Nevertheless, the research was innovative in demonstrating that psychiatric phenomena could be linked to electrophysiological markers of brain activity during sleep, offering critical evidence for the biological basis of mental illness.

Interestingly, the first polygraphic sleep study in patients with affective disorders predated the discovery of REM sleep. Conducted in 1946 by Diaz‐Guerrero et al., the study examined bipolar depression and noted ‘a greater proportion of sleep which is light and more frequent oscillations from one level of sleep to another than normally occurs’ (Diaz‐Guerrero et al. 1946). However, subsequent studies revealed considerable variability in sleep features among patients. For instance, some individuals displayed signs of hypersomnia rather than insomnia (Kupfer et al. 1972). Other findings once thought specific to depression—such as shortened REM latency (Kupfer and Foster 1972)—were also found to lack diagnostic specificity and were not consistently present across all patients. These varying results helped shape the current view of psychiatric conditions as overlapping, transdiagnostic syndromes involving emotional and cognitive dysregulation. Genetic studies have since reinforced these observations, showing shared genetic influences among psychiatric diseases, for example, schizophrenia and bipolar disorder, as well as between sleep traits and psychiatric conditions such as insomnia and depression (Lane et al. 2017).

Several papers have acclaimed the German psychiatrist Johann Christian August Heinroth (1773–1843)—the first university professor of psychiatry—as a pioneer of sleep deprivation in the treatment of ‘melancholia’ (Steinberg and Hegerl 2014). The antidepressant effects of total sleep deprivation were later confirmed (Pflug and Tolle 1971), and laid the foundation for specific treatment protocols—some of which are still in use today, even though their exact mechanisms remain under investigation. Advances in circadian rhythm research furthered the therapeutic possibilities. Rosenthal et al. observed that reduced daylight exposure during winter contributed to depressive symptoms in some individuals, which could be alleviated with exposure to bright artificial light (Rosenthal et al. 1984). This finding marked the formal introduction of light therapy as a treatment for seasonal affective disorder, and more recently, also for non‐seasonal depression (Tao et al. 2020).

Research into schizophrenia was initially motivated by the resemblance between daytime hallucinations in psychosis and hallucinatory dream experiences. However, the anticipated abnormalities in REM sleep among patients with schizophrenia were not consistently observed. In fact, Dement and others found in early polygraphic studies that REM periods were comparable between patients and healthy controls (Dement 1955). A reduction in slow‐wave sleep in schizophrenia has been reported, but this finding lacks specificity (Igert and Lairy 1962). Using high‐density EEG montages, researchers at the University of Wisconsin demonstrated a reduction in centroparietal EEG power in specific frequency bands, along with decreased sleep spindle activity in schizophrenia (Ferrarelli et al. 2007). These neural changes are associated with cognitive impairments and are considered endotypic features of the disorder.

Although sleep disturbances have been described in psychiatric illness for centuries, and were already mentioned in ancient accounts of melancholia, they only emerged into formal diagnostic criteria for psychiatric research in the 1970s (Feighner et al. 1972). The DSM‐III formally included insomnia or hypersomnia as diagnostic criteria for mood disorders and recognised reduced need for sleep as a core symptom of mania in bipolar disorder (APA 1980). The critical role of sleep in mental health became even more evident through late 20th‐century epidemiological studies, which showed that insomnia often precedes depression (Baglioni et al. 2011; Ford and Kamerow 1989). These findings spurred interest in treating insomnia as a preventative strategy. Contemporary research has since demonstrated the efficacy of CBT‐I in patients with co‐occurring mental disorders, particularly depression, posttraumatic stress disorder, and alcohol dependence (Hertenstein et al. 2022).

Today, sleep disturbances and psychiatric disorders are widely recognised as bidirectionally related (Baglioni et al. 2016; Riemann et al. 2020). Sleep problems can contribute to the onset, persistence, and recurrence of psychiatric disorders, while psychiatric conditions can likewise cause sleep disturbances. In some cases, the two may also occur independently. By eliminating the distinction between primary and secondary sleep disorders, the DSM‐5 acknowledges sleep disorders as independent conditions that warrant separate diagnosis and treatment, even when psychiatric comorbidities are present.

### 1.5 Sleep‐Disordered Breathing

Although previous cases had been published—some noting breathing pauses during sleep—a case report by Burwell et al. was key in establishing the link between morbid obesity, hypersomnolence, and sleep‐disordered breathing (SDB) (Burwell et al. 1956). This report provided a detailed clinical account and introduced the term Pickwickian Syndrome, drawing a parallel between the patient's characteristics and *Joe the Fat Boy*, a character from Charles Dickens' novel *The Pickwick Papers*. While Burwell et al. observed snoring, they did not report episodes of breathing cessation during sleep. Instead, they attributed the hypersomnolence to alveolar hypoventilation with hypercapnia—all of which resolved with weight loss.

Subsequent studies, primarily by European researchers, included both daytime and nocturnal sleep analyses of Pickwickian patients, and revealed periodic respiration with frequent apnoeic episodes (Lavie 2008). Initially, hypersomnolence was thought to result from ‘carbon dioxide poisoning’, but researchers soon recognised that apnoea was the primary cause. This was confirmed when tracheostomy led to the immediate normalisation of hypersomnolence and respiratory blood gases, demonstrating that sleep‐related upper airway obstruction was responsible for both disrupted breathing and excessive daytime sleepiness.

At Stanford University, Christian Guilleminault and colleagues expanded this understanding by demonstrating that sleep apnoea, even in the absence of obesity, was linked to a broad range of clinical symptoms. In their seminal publication ‘The Sleep Apnea Syndromes’, they made a comprehensive description of signs, symptoms, and laboratory findings in obstructive sleep apnoea (OSA) (Guilleminault, Tilkian, et al. 1976). They recommended that the diagnosis should be considered when at least 30 apnoeic episodes could be observed during 7 h of sleep. This work was pivotal as it provided evidence that previously unexplained medical conditions stemmed from OSA, which from then on was established as a distinct medical disorder.

While OSA can lead to severe, potentially life‐threatening complications, treatment options in the 1960s and 1970s were limited to weight loss and tracheostomy. A breakthrough came in 1981 when Sullivan et al. introduced continuous positive airway pressure (CPAP) as a treatment for OSA (Sullivan et al. 1981). This revolutionary therapy induced complete resolution of respiratory disturbances and immediate symptom relief, even in the most severe cases. It quickly became the gold standard for OSA management. The success of CPAP not only prompted the mass production of CPAP devices but also accelerated the use of polysomnography for diagnostic purposes of OSA and other common sleep disorders. Given the high prevalence of OSA, sleep laboratories and multidisciplinary sleep centres mushroomed worldwide.

However, as CPAP therapy can be cumbersome and poorly tolerated by some patients, alternative treatments had to be developed. Pharyngoplasty, initially introduced to treat snoring (Fujita et al. 1981), often proved unreliable in OSA management (Epstein et al. 2009). Mandibular advancement devices (MRAs) have since become the most commonly used CPAP alternative (Schmidt‐Nowara et al. 1995). While MRAs are less efficacious than CPAP in reducing the apnoea–hypopnoea index (AHI), they appear equally effective in improving symptom scores (Ramar et al. 2015).

Over the past decade, research has highlighted the complex pathophysiology of OSA, revealing that multiple factors contribute to its development (Eckert et al. 2013). This growing understanding has paved the way for personalised treatment strategies that attempt to target specific endotypic traits in individual patients (Edwards et al. 2019).

### 1.6 Paediatric Sleep Disorders

Sleep disorders in children remained largely overlooked in medical literature until the 1970s. By the 1980s, paediatric textbooks began addressing sleep disturbances as a distinct area of interest within paediatrics. Since then, the field has gained increasing recognition, leading to the publication of numerous medical and public books. However, it was not until 1995 that the first comprehensive textbook, *Principles and Practice of Paediatric Sleep Medicine* was published (Ferber and Kryger 1995). This milestone helped establish paediatric sleep medicine as an independent subdiscipline, often developing under the broader framework of adult sleep medicine. As interest grew among both general and specialised paediatricians, dedicated paediatric sleep centres began to emerge.

At the turn of the 21st century, sleep research expanded across multiple medical and social health fields, revealing distinct differences between paediatric and adult sleep disorders. Population studies highlighted the health risks associated with declining sleep duration in modern societies, particularly among children. Observational research in paediatric sleep physiology and pathophysiology led to key discoveries. For example, the identification of rapid eye movements in infants during paradoxical sleep—characterised by increased motility, irregular breathing, and elevated heart rate—contributed to the understanding of REM sleep, later confirmed in adults (Aserinsky and Kleitman 1953). Meanwhile, studies on NREM sleep demonstrated its role in typical paediatric parasomnias, which originate from slow‐wave sleep rather than REM sleep as previously surmised (Broughton 1968).

Yoss and Daly provided the first systematic description of narcolepsy in children aged 3 to 14 years and emphasised the frequent misdiagnosis of the disorder (Yoss and Daly 1957).

Sleep was also linked to mortality risks such as sudden infant death syndrome (SIDS), which accounts for nearly one‐third of deaths in infants aged 1–6 months. The research of André Kahn on respiratory events during sleep in infants was fundamental. He was among the first authors to observe apnoeas during sleep in future SIDS victims (Kahn 1979). He pioneered the development of ambulatory monitoring systems to detect respiratory spells in infants (Kahn and Blum 1982). Yet, despite extensive research, the precise causes of SIDS are not fully elucidated (AAP 1992).

Paediatric OSA was initially described by Guilleminault, Eldridge, et al. (1976). In a subsequent report of 50 patients, it was shown that the clinical phenotype of OSA in children is different from that in adults (Guilleminault et al. 1981). While excessive daytime sleepiness is a key symptom in adults, children with OSA or narcolepsy often present with hyperactivity or attention deficits instead (Chervin et al. 2002). Research on paediatric OSA has evolved over time, initially focusing on its impact on growth and development in the 1980s, shifting towards cognitive and behavioural concerns in the 1990s, and more recently exploring its associations with obesity, inflammation, and biomarkers (Bruni and Ferri 2015). Similarly, paediatric RLS, first described in 1994 (Walters et al. 1994), differs from the adult form. In children, RLS is often linked to growing pains and attention‐deficit hyperactivity disorder (ADHD), necessitating the development of specialised diagnostic criteria (Picchietti et al. 2013). These distinctions underscore the unique characteristics of paediatric sleep disorders despite their similarities to adult conditions. In recognition of these differences, dedicated paediatric sections have been incorporated into the ICSD (AASM 2023).

Paediatric sleep medicine continues to thrive, driven by the unique nature of sleep throughout human development. Scientific advances in the field have been propelled by numerous researchers over the past decades (Bruni and Ferri 2015). Currently, PubMed records between 3000 and 4000 annual publications on sleep in children aged 1–18 years. The field's growth is also reflected in the establishment of specialised scientific societies. The European Paediatric Sleep Club (EPSC) was founded in 1991 under the umbrella of the European Sleep Research Society (ESRS). Thanks to the efforts of André Kahn and the pivotal contributions of Christian Guilleminault, the EPSC later evolved into the International Paediatric Sleep Association (IPSA), which was officially established during the World Association of Sleep Medicine meeting in Berlin on October 13, 2005 (Bruni and Ferri 2015).

Through ongoing research and collaboration, paediatric sleep medicine continues to expand, offering deeper insights into childhood sleep disorders and their long‐term health implications.

## 2 What Is Sleep Medicine?

Sleep medicine is part of a broader group of cross‐sectional disciplines that are grounded in shared biological processes and integrate multiple organ systems within a specific context. Similar ‘horizontal’ disciplines have emerged in areas such as age‐related care (e.g., paediatrics and geriatrics), cell biology (e.g., oncology), and microbiology (e.g., infectiology), among others. Sleep, as a fundamental biological process, can be disrupted by a wide range of disease mechanisms. Evidently, the various sleep disorders have a common ground underpinning the concept of clinical sleep medicine as we know it today. As an inherently interdisciplinary field, sleep medicine is accessible to professionals from diverse educational and professional backgrounds.

Comprehensive, multidisciplinary curricula have been developed, elevating sleep medicine to the same educational standards as those upheld in other established medical disciplines (Grote et al. 2022; McNicholas et al. 2025; Penzel et al. 2021). Sleep medicine roughly encompasses knowledge and practice based in general medicine, respiratory medicine, neurology, circadian rhythm biology, psychiatry and psychology, dentistry and ear, nose and throat surgery, across the lifespan, thereby incorporating both the paediatric and adult fields. Sleep disorders in themselves co‐occur relatively frequently, for example, OSA and insomnia disorder, narcolepsy type 1 and OSA, periodic limb movements and RBD. Therefore, it is important that sleep clinicians have knowledge about all sleep disorders and their potential interactions.

Some countries have been at the forefront of adopting sleep medicine as a subspecialty. Full recognition has occurred in Germany and France, among others. In most European countries, however, sleep medicine remains out of scope as a separate discipline. National and international bodies have established specific educational strategies as well as advances in the field through the publication of classifications and comprehensive guidelines.

The most widely used nosological standard in sleep medicine is the International Classification of Sleep Disorders (ICSD). First released in 1979 by the American Association of Sleep Disorders Centers (ASDC 1979), the ICSD has been periodically revised to reflect advances in scientific understanding and clinical practice. The third edition, published by the AASM in 2014, was recently updated with a text revision (ICSD‐3‐TR) (AASM 2023). However, this revision involved only minor editorial changes and did not include substantial modifications to the classification itself. The 11th revision of the International Statistical Classification of Diseases and Related Health Problems (ICD‐11) includes a separate chapter dedicated to sleep–wake disorders (WHO 2022). This chapter systematically outlines the major categories of sleep disorders and highlights their clinical relevance. Its introduction underscores the growing recognition of sleep disorders as a major component of the global burden of disease. As to guidelines, the ESRS has published a European Catalogue of Knowledge and Skills in which requirements for education and training in sleep medicine are stipulated (Penzel et al. 2021, 2014).

Because detailed investigations are often necessary to detect organic sleep disorders, sleep medicine has led to major evolution in many areas. The development of technology to record sleep has led to the development of specialised sleep units with highly trained technicians and nurses. Evolutions in the management of sleep disorders have also transformed nursing, physiology, physiotherapy, and psychology with the introduction of CPAP for OSA and CBT for insomnia.

Table 2 summarises the diagnostic and management strategies applied to sleep disorders in terms of what is currently known, including unresolved areas and areas of uncertainty (AASM 2023).

**TABLE 2:** Diagnostic criteria for sleep disorders classified according to the ICSD‐3‐TR.

Sleep disorder	Clear diagnostic criteria established	Presence of reliable biomarker	Fulfils criteria to be classified as a disease/disorder	Uncertainties and unresolved issues
**Insomnia**				
Chronic insomnia disorder	Symptom based	No	Yes	Pathophysiology still unclear
Short‐term insomnia disorder	Symptom based	No	Yes	
Other insomnia disorder				
**Sleep‐related breathing disorders**				
**Obstructive sleep apnoea disorders** Obstructive sleep apnoea, adult Obstructive sleep apnoea, paediatric	Polysomnography (PSG) with specific thresholds for apnea‐hypopnea index (AHI). Symptoms such as habitual snoring, restless sleep, and daytime behavioural issues.	Apnoea‐hyopnoea index—but this varies. Can be used to assess response to treatment Sleepiness is measured most frequently using the ESS but also elicited on history.	Yes	Age and sex‐ related variation of AHI index and of clinical symptoms Clear definition of OSA phenotypes Management for mild cases or non‐surgical candidates.
**Central Sleep Apnoea Disorders** CSA + Cheyne‐Stokes Respiration CSA due to a medical disorder without Cheyne‐Stokes Respiration CSA due to high‐altitude periodic breathing Primary CSA Primary CSA of Infancy Primary CSA of Prematurity Treatment‐emergent CSA	Polysomnography (PSG) with specific thresholds for apnea‐hypopnea index (AHI). Symptoms can be highly variable.	As above.	Yes	Ongoing research into appropriate management based on pathophysiology.
**Sleep‐related Hypoventilation Disorders** Obesity hypoventilation syndrome Congenital central alveolar hypoventilation Late‐onset central hypoventilation with hypothalamic dysfunction Idiopathic central alveolar hypoventilation Sleep‐related hypoxaemia disorder	Polysomnography (PSG) with specific thresholds for apnea‐hypopnea index (AHI). Symptoms such as habitual snoring, restless sleep, and daytime behavioural issues.	As above. Yes = PHOX2b mutations	Yes Yes	Yes. Non‐invasive ventilation with or without oxygen.
Narcolepsy Type 1	Yes	Yes	Yes	Detailed pathophysiology, disappearance or silencing of hypocretin neurones, new treatments
Narcolepsy Type 2	No	No	No	Heterogeneous clinical group of patients (rare early NT1), role of insufficient sleep often not excluded correctly, lack of consistent MSLT results over time. Pathophysiology unknown. Probably multiple pathways
Idiopathic hypersomnia (with normal sleep time)	No	No	No	Very heterogeneous group of patients, insufficient sleep or irregular sleep wake cycle, often not excluded correctly, lack of consistent MSLT results over time. Relatively rare in our experience, probably because we insist on 14 days of actigraphy before 24 h PSG. Pathophysiology unknown
Idiopathic hypersomnia (with long sleep time)	Yes (but see later)	No	Yes (but see later)	Hard to diagnose. Ad libitum sleep to establish sleep need is practically difficult to achieve. The 660‐min limit is too strict but no consensus on other limit. Pathophysiology unknown
Kleine‐Levin syndrome	Clinical	No	Yes	Defined by symptom criteria, easy in typical cases but difficult for atypical cases. Pathophysiology unknown
**Circadian rhythm sleep–wake disorders**				
Delayed sleep–wake phase disorder	Yes	Can do accurate and quantitative 24 h dosing of melatonin Alternative is core temperature monitoring, also only used for research	Yes	Diagnosis of disorder based on patterns of sleep and wakefulness using actigraphy. Could more accurate monitoring be developed?
Advanced sleep–wake phase disorder	Yes		Yes	
Irregular sleep–wake rhythm disorder	Yes		Yes/no	
Non‐24‐h sleep–wake Rhythm disorder	Yes	No	Yes	
Shift work disorder	Yes/no	No	Probably	
Jet lag disorder	Yes		Yes	
**Parasomnias**				
**NREM‐related parasomnias** Confusional arousals Sleepwalking Sleep terrors Sleep‐related eating disorder Sexsomnia?	Yes, clinical	No	Yes	Differential diagnosis with other phenomena (i.e., seizures); Particularly difficult in the context of psychological and psychiatric disorders, for example, nocturnal dissociation
**REM‐related parasomnias** REM sleep behaviour Disorder	Yes	Yes/No	Yes	Diagnosis using markers associated with synucleinopathy are potentially useful, but secondary RBD associated with many other disorders
Recurrent isolated sleep Paralysis	No	No	Yes	Associated commonly with anxiety
Nightmare disorder	No	No	Yes	Subjective assessment
**Sleep‐related movement disorders**				
Restless Legs syndrome	Clinical criteria	No	Yes	RLS is easy for clear cut cases, difficult when co‐morbidities; PLMS detected on overnight studies do not necessary correlate with symptoms. Often occurs in the context of OSA and also with anxiety. Clinical significance PLMD under debate. Reasons for persistence into adulthood unknown.
Periodic limb movement disorder	Yes	Yes	Yes‐but clinical significance/relevance as cause of clinical symptoms such as EDS under debate.	
Nocturnal muscle cramps	Clinical criteria	No	?	
Sleep‐related bruxism	Clinical criteria and EMG on PSG	Yes	?	
Sleep‐related rhythmic movement disorder	Yes. Clinical and vPSG criteria	Yes	Yes	
Benign sleep myoclonus of infancy	Clinical criteria	No	?	
Propriospinal myoclonus at sleep onset	Clinical criteria	No	?	

*Note*: Isolated symptoms and normal variants have been excluded as have disorders secondary to medication or substance, another medical condition or mental disorder.

## 3 Clinical Pathways in the Management of Sleep Disorders

There are a number of clinical pathways for managing sleep disorders depending on the type of sleep disorder suspected, facilities required for diagnosis and for management. In turn, this depends on resources available locally and nationally, funding and reimbursement practices, and recognition of the type of sleep disorder in the first place (Fietze et al. 2022). Table 3 summarises the strengths and weaknesses of our current state of knowledge regarding specific management strategies for sleep disorders.

**TABLE 3:** Management of sleep disorders classified according to the ICSD‐3‐TR.

Sleep disorder	Management well established	Management based on randomised controlled trials	Uncertainties and unresolved issues
**Insomnia**			
Chronic insomnia disorder	Yes	Yes	CBT and comorbidities, third generation techniques, long term treatment with hypnotics, combination therapies
Short‐term insomnia disorder	Yes	Yes	
Other insomnia disorder			
**Sleep‐related breathing disorders**			
**Obstructive sleep apnoea disorders**			
Obstructive sleep apnoea, adult	Yes	Yes	Unresolved questions regarding impact of management on co‐morbidities; practical value of endotyping.
Obstructive sleep apnoea, Paediatric	Yes, adenotonsillectomy CPAP for selected cases Not for mild and non‐surgical cases	Yes	Residual OSA post‐adenotonsillectomy Optimal treatment for mild OSA Adherence to CPAP
**Central sleep apnoea disorders**			More detailed studies necessary to phenotype accurately; treatment not finalised.
CSA + Cheyne‐Stokes Respiration	Yes	Yes	
CSA due to a medical disorder without Cheyne‐Stokes respiration	No	No	
CSA due to high‐altitude periodic breathing	Yes	No	
Primary CSA	Yes	No	
Primary CSA of Infancy	Yes	No	
Primary CSA of prematurity	Yes	No	
Treatment‐emergent CSA	Yes	No	
**Sleep‐related hypoventilation disorders**			
Obesity hypoventilation syndrome	Yes	Yes	
Congenital central alveolar hypoventilation	Yes	No	
Late‐onset central hypoventilation with hypothalamic dysfunction	Yes	No	
Idiopathic central alveolar hypoventilation	Yes	No	
Sleep‐related hypoxaemia disorder	Yes	No	
**Central disorders of hypersomnolence**			Non pharmacological management. Future role of orexin agonists?
Narcolepsy Type 1	Yes	Yes	
Narcolepsy Type 2	No/Yes	Yes/no	
Idiopathic hypersomnia	Yes	No/Yes	
Kleine‐Levin Syndrome	Yes	No	
**Circadian rhythm sleep–wake disorders**			Use of melatonin is variable and local recommendations vary
Delayed sleep–wake phase disorder	Yes but variable dosing	?	
Advanced sleep–wake phase disorder	No	?	
Irregular sleep–wake rhythm disorder	?	?	
Non‐24‐h sleep–wake Rhythm disorder	Yes but variable dosing	Yes	
Shift work disorder	?	No	
Jet lag disorder	Yes but variable dosing	No	
**Parasomnias**			
**NREM‐related parasomnias** Confusional arousals Sleepwalking Sleep Terrors Sleep‐related Eating disorder Sexsomnia	Yes, sleep hygiene, scheduled awakenings (not proven to work), drugs, recognising periods of excessive stress and anxiety which can exacerbate behaviours	No.	No RCTs for paediatric and adult populations Uncertain role of pharmacotherapy Underlying psychological issues need to be addressed and often are not
**REM‐related parasomnias** REM sleep behaviour disorder	Yes	No	Role of managing trauma unknown; potential for disease‐modifying agents for alpha‐synucleinopathies in iRBD.
Recurrent isolated sleep paralysis	No	No	Empirical treatments only
Nightmare disorder.	Guidelines in place, and treatment is empirical	Limited to psychological therapies	Drug treatments empirical. Treatments for psychological trauma, for example, EMDR might be useful
**Sleep‐related movement disorders**			
Restless legs syndrome	Yes	Yes	Pathophysiology currently incompletely understood which limits treatment options Can be extremely difficult to treat and often does not resolve when presenting into adulthood
Periodic limb movement disorder	Yes‐but clinical significance not clearly established	No	
Nocturnal muscle cramps	Yes—but empirical	No	
Sleep‐related bruxism	Protection of teeth using a splint; anxiolytic medications; botox	No	
Sleep‐related rhythmic movement disorder	No	No	
Benign sleep myoclonus of infancy	No	No	
Propriospinal myoclonus at sleep onset	No	No	

*Note*: Isolated symptoms and normal variants have been excluded as have disorders secondary to medication or substance, another medical condition or mental disorder.

Sleep disorders occur very commonly within the population, both in children and adults. Those arising as a result of poor lifestyle, for example, caffeine abuse, behavioural sleep restriction, irregular bedtimes, can generally be managed in a primary care setting using basic sleep hygiene principles. The commonest sleep issue in the community is behaviourally induced sleep insufficiency (Ohayon 2008; Ohayon et al. 2012).

The ideal pathway for a patient with a suspected organic sleep disorder is to be referred to a sleep disorders centre where a clinician trained in the specialty can take a history, perform a clinical exam and determine the appropriate investigations, followed by the appropriate management decisions based on all of the available information. However, many centres in Europe and around the world grew organically over time arising from different specialities. As a result, different services often focus either primarily on what are traditionally considered neurological disorders or on disorders that are deemed respiratory conditions. Given the frequent co‐occurrence of sleep and circadian disorders, often compounded by psychological factors, multidisciplinary sleep centres that provide comprehensive assessment and management should be the standard. Some basic conditions, for example, insomnia without co‐morbid sleep disorders can be managed in a stand‐alone manner in a variety of settings by CBT‐I practitioners, but again, care should be integrated and the possibility for further specialised assistance should be available.

Investigations have traditionally been based on what has always been considered the ‘gold standard’, namely polysomnography (PSG) combining EEG, EOG, EMG and cardiorespiratory recording with monitoring of body position and audiovisual input (Rundo and Downey 2019) (Gauld and Micoulaud‐Franchi 2021). Polygraphy, a pared down version of PSG using a limited montage (e.g., type III devices that lack neurophysiological signals and video) is used for suspected sleep‐related breathing disorders (Riha et al. 2023). While it has good positive predictive value in patients with a high clinical suspicion of OSA, it cannot rule out OSA in a more complex context, especially when comorbid insomnia is present. Over time, and in the context of technological advances, the number of signals derived from sensors applied to the body has increased (Collop et al. 2011). Contactless technologies are being developed that hold promise for further simplification of diagnostic processes. As with the classification of sleep disorders discussed above, the scoring of these signals has undergone a number of iterations and attempts at standardisation, with the AASM principally involved in their creation (Berry et al. 2012; Iber et al. 2007). This has enabled the development of reciprocal, internationally accepted guidelines that promote consistency in disease definitions—particularly important given the critical role of overnight studies in diagnosis—and ensures both safe, appropriate management and the effective direction of research. An overview of techniques for acquiring signals overnight is summarised in a recent publication assessing limited channel sleep studies with the pros and cons outlined (Riha et al. 2023).

The growing impetus to incorporate artificial intelligence (AI) in various aspects of medicine also affects sleep disorders. A task force set up by the AASM outlines five key areas for the application of big data in sleep medicine: improved diagnostic classification and accuracy; predictive treatment models; subtyping sleep disorders; the automation of sleep scoring; and patient‐centred approaches to improve treatment compliance, such as with PAP therapy (Goldstein et al. 2020). In advancing these goals, it is imperative to ensure the responsible use of AI and big data, to address underfitting or overfitting of various models developed, and to maintain patient privacy. The latter has been discussed in several recent overviews on the subject (Alattar et al. 2024; Bandyopadhyay and Goldstein 2023; Sun et al. 2025).

Large strides have been made in telemonitoring and management through cloud‐based systems, particularly in the treatment of sleep‐disordered breathing. Real‐time tracking of adherence, mask leaks, and pressure settings in CPAP therapy—enabled by integrated cloud‐based technologies—offers clear advantages.

In summary, appropriate clinical pathways rely on a multidisciplinary, integrated approach offered in dedicated sleep centres. While traditional diagnostic tools like PSG remain foundational, technological advances, including simplified sensors, contactless monitoring, and AI‐driven solutions, are reshaping diagnostics and management. Consistent classification systems and responsible use of emerging technologies, including big data and telemonitoring, are essential to improving diagnostic accuracy, treatment personalisation, and long‐term outcomes in sleep medicine.

## 4 Current Issues in Sleep Medicine

### 4.1 Generic Issues in Sleep Medicine

Despite the universal relevance of sleep and the huge prevalence of sleep disorders in the population, sleep medicine all too often faces marginalisation and poor access to resources, particularly in terms of access to and funding of healthcare as well as clinical research.

A major cultural hurdle within the field is the misconception that, because everyone sleeps, everyone understands sleep. This oversimplification undermines the expertise required to manage sleep disorders and perpetuates misinformation. The rise of self‐proclaimed sleep consultants and social media influencers further complicates the landscape. These unregulated sources often promote oversimplified or misleading advice, creating confusion among patients and undermining evidence‐based care.

Recent years have also seen growing reliance on consumer‐grade and portable diagnostic tools. While these technologies can increase access to the measurement of sleep variables, they are limited by a lack of critical features such as neurophysiologic signal recording needed for accurately measuring sleep stages and detecting subtle but clinically significant disturbances. Conversely, indications of disturbed nocturnal sleep may cause unnecessary health concerns in people without sleep complaints (Baron et al. 2017). Direct‐to‐consumer apps and over‐the‐counter devices bypass clinical interpretation, reducing sleep medicine to a consumer service model. This trend not only diminishes the role of qualified specialists but also weakens the multidisciplinary approach necessary for accurate diagnosis and effective treatment.

Several areas within sleep medicine remain marked by uncertainty and conceptual blind spots. While several sleep disorders retain diagnostic validity when viewed through robust theoretical frameworks, such as Koch's postulates (Hucklenbroich 2014; Spitzer et al. 1978) and the Bradford‐Hill criteria (Hill 1965), others rest on less secure foundations. Some paradigms endure unchallenged for extended periods, potentially impeding progress. As such, emerging models that question traditional assumptions should be welcomed. The field still lacks tried‐and‐tested biomarkers to support many presumed diagnoses, and their discovery remains a critical goal. This ongoing search for stronger, evidence‐based disease models is reflected in the subsequent updates of the ICSD described above.

Pharmacological therapies for sleep disorders have largely emerged from empirical observations rather than rigorous trials. For many conditions, such as RBD and other parasomnias, randomised controlled trials (RCTs) are lacking, often due to funding challenges. Even when RCTs exist, they rarely exceed 1 year in duration, and data on long‐term side effects remain limited. Treatment guidelines often rely on placebo‐controlled studies rather than comparisons with existing, cost‐effective alternatives, particularly for newer agents targeting residual sleepiness in OSA or central hypersomnolence disorders (Dauvilliers et al. 2013). These gaps hinder the development of a robust, evidence‐based pharmacological framework adaptable to diverse healthcare systems (Bassetti et al. 2021). An additional, underexplored concern is bioequivalence after drug patent expiration. Anecdotal reports suggest that some generic formulations of drugs, such as modafinil or sodium oxybate, may be less effective or safe than brand‐name versions. Similar issues have been better documented in other fields like cardiology, epilepsy, and transplantation medicine (Dauvilliers et al. 2022; Lechat 2022).

### 4.2 Insomnia Disorder

A significant barrier in the management of chronic insomnia is limited engagement with professional healthcare services. Epidemiological data suggest that fewer than 20% of individuals suffering from chronic insomnia actively seek medical attention (Chalet et al. 2024). The majority remain undiagnosed and untreated or instead rely on non‐clinical interventions such as internet‐based resources, self‐help literature, or over‐the‐counter medications obtained through pharmacies or alternative sources.

This presents a clinical concern, as proper evaluation through differential diagnosis is essential in cases of chronic insomnia. Evidence‐based treatment options—both pharmacological and psychotherapeutic—are well established. However, when these are bypassed, patients are at increased risk for developing comorbid psychiatric conditions, particularly anxiety and depressive disorders (Baglioni et al. 2011; Hertenstein et al. 2019).

Given the established association between chronic insomnia and poor mental health outcomes, it is reasonable to assume that early and effective treatment may mitigate these risks (Boland et al. 2023). Thus, raising awareness about insomnia as a serious clinical condition is critical—not only among the general public but also within the healthcare system itself.

To address the problems of diagnosis and access to appropriate treatment, recent work has proposed a stepped‐care model for the diagnosis and management of insomnia, offering a scalable framework that aligns clinical resources with patient needs (Baglioni et al. 2023). This approach emphasises initial low‐intensity interventions (e.g., dCBT‐I) followed by more specialised care for non‐responders, thereby improving access while preserving clinical efficacy.

For clinicians, adopting structured, evidence‐based care pathways and promoting early recognition of insomnia as a treatable disorder can significantly improve outcomes and reduce the long‐term burden of comorbid mental health conditions.

Currently, no medications have been approved by the U.S. Food and Drug Administration (FDA) or the European Medicines Agency (EMA) for the long‐term treatment of chronic insomnia disorder. While most chronic medical conditions have access to ‘on‐label’ pharmacological therapies, the lack of approved long‐term treatments for insomnia represents a significant gap in the therapeutic landscape. Due to the risks of tolerance and adverse effects, benzodiazepines and benzodiazepine receptor agonists are not recommended for use beyond 4 weeks (Riemann et al. 2023). As a result, long‐term pharmacological management often relies on ‘off‐label’ use of medications such as antidepressants or antiepileptics (Dujardin et al. 2022).

The introduction of dual orexin receptor antagonists (DORAs) marks a notable recent advancement in insomnia pharmacotherapy (Mignot et al. 2022; Riemann et al. 2023). Among these, daridorexant is the only compound thus far approved by the EMA. In a clinical trial, daridorexant at a 50 mg dose was shown to be safe and well tolerated, with sustained improvements in both sleep parameters and daytime functioning after 12 months of continuous use (Kunz et al. 2023). Nonetheless, further RCTs will be needed before DORAs can be formally approved for long‐term treatment of chronic insomnia disorder.

### 4.3 Neurological Sleep Disorders

#### 4.3.1 Narcolepsy and Other Disorders of Central Hypersomnolence

Chronic sleep deprivation is a prevalent contributor to EDS, frequently resulting from lifestyle factors, shift work, or high‐demand occupational schedules (Ohayon 2008). In industrialised regions, a substantial proportion of the population experiences insufficient sleep on a regular basis, exacerbating daytime cognitive and functional impairments. In clinical settings, distinguishing chronic sleep deprivation from primary sleep disorders associated with EDS remains a diagnostic challenge.

At the individual level, establishing whether EDS stems from chronic sleep insufficiency is critical. These cases are potentially reversible through behavioural and lifestyle interventions, primarily sleep extension (Baumann‐Vogel et al. 2021). From a public health perspective, the implications of widespread chronic sleep deprivation are profound, contributing to increased risk of accidents, social dysfunctioning, and possibly heightened vulnerability to mood disorders. Development of objective, easily accessible biomarkers or screening criteria could support early identification and education to mitigate sleep deprivation across populations.

The diagnostic framework for idiopathic hypersomnia (IH) remains complex and controversial. Current diagnostic criteria are not fully evidence‐based, are difficult to apply consistently, and may exclude individuals with debilitating symptoms from receiving a formal diagnosis. The lack of reliable biomarkers and significant phenotypic overlap with narcolepsy type 2 further complicates diagnostic clarity. Whether IH and narcolepsy type 2 represent distinct entities remains a subject of ongoing debate (Fronczek et al. 2020; Gool et al. 2022; Lammers et al. 2020).

A critical question in clinical practice is the reliability and utility of the Multiple Sleep Latency Test (MSLT) as the diagnostic gold standard for central hypersomnolence disorders. The MSLT is resource‐intensive, highly sensitive to prior sleep deprivation, and often yields variable results over time in individuals with central hypersomnolence, exceptions being patients with narcolepsy type 1, where hypocretin deficiency offers a clear pathophysiological correlate and more robust diagnostic consistency (Lammers et al. 2020). This underscores the need for alternative or complementary objective measures of EDS, to define and identify ‘real’ distinct disorders in those currently diagnosed with narcolepsy type 2 or IH (Gool et al. 2022).

To date, causal mechanisms have been clearly defined only in narcolepsy type 1, enabling disease‐specific therapeutic strategies. In contrast, other central disorders of hypersomnolence lack defined pathophysiological substrates, limiting treatment to symptomatic management.

Finally, there is a notable lack of health‐economic data quantifying the societal and healthcare burden of both sleep deprivation‐induced and pathological EDS in primary sleep disorders. Robust health‐economic studies are needed to assess the long‐term costs, productivity losses, and healthcare resource utilisation associated with these conditions—information that could guide policy and resource allocation for sleep health initiatives.

#### 4.3.2 Restless Legs Syndrome and Periodic Limb Movement Disorder

Managing restless legs and periodic leg movement disorders is challenging due to limited disease recognition, absence of a diagnostic test, unclear pathophysiology, and inconsistent management practices. While scoring criteria for nocturnal leg movements exist (Berry et al. 2012), their management without RLS remains uncertain. Dopamine agonists, though effective, risk augmentation syndrome with long‐term use (Allen et al. 2014), complicating future treatments (Garcia‐Borreguero et al. 2019). Alternatives may reduce this risk but may be less effective and may have side effects. Current recommendations reflect these uncertainties as indicated by disagreements between American and European guidelines (Trenkwalder et al. 2024; Winkelman et al. 2025). Non‐pharmacological approaches are under investigation as promising alternatives.

#### 4.3.3 REM Sleep Behaviour Disorder

The causative mechanisms of RBD are largely unknown (Maya et al. 2024). Additional treatment options are needed for refractory patients. There is a lack of trials with substances that potentially inhibit the emergence or progress of neurodegenerative disease manifestations (Stefani et al. 2025).

### 4.4 Sleep and Psychiatric Disorders

Psychological and psychiatric factors may play a contributing role in clinical manifestations of sleep disorders in general, in addition to the overlay of medication side‐effects, pain, other comorbid medical conditions, and poor sleep hygiene. It is key for the sleep specialist to be able to assess the importance of all these factors when teasing out treatment strategies to manage disturbed sleep.

Sleep disturbances are both a cause and consequence of psychiatric disorders (Freeman et al. 2020); however, no unified theoretical framework currently exists to explain the bidirectional and disorder‐specific interactions between sleep and psychopathology. It also remains unclear which individuals with sleep disturbances are at increased risk of developing psychiatric conditions, and conversely, which psychiatric presentations are most likely to precipitate chronic sleep dysfunction.

Identifying biomarkers linked to specific psychiatric disorders or underlying neuropsychological traits could enable both early intervention and the development of personalised treatment strategies. Moreover, tailoring psychiatric pharmacotherapy based on individual sleep–wake profiles may enhance treatment efficacy and reduce adverse effects (Paunio 2021).

Despite growing evidence of the clinical relevance of sleep in psychiatric populations, a major limitation lies in the disconnect between research and clinical practice. In most psychiatric care settings, sleep and circadian rhythm disturbances are not systematically assessed, monitored, or integrated into treatment planning. As a result, the full therapeutic potential of evidence‐based sleep interventions—such as CBT‐I or chronotherapeutic approaches—remains underutilised in routine care.

Closing this gap will require greater integration of sleep assessment into psychiatric diagnostics, education of mental health professionals on the relevance of sleep in psychopathology, and broader implementation of targeted, sleep‐focused interventions (Martin et al. 2024).

### 4.5 Sleep‐Disordered Breathing

The prevailing paradigm in OSA is that recurrent apnoeas and hypopnoeas lead to systemic disease manifestations in a dose‐dependent manner (AASM 2023). Conventionally, the AHI has been used to categorise OSA disease severity as mild, moderate, or severe (AASM 1999). However, recent evidence has highlighted significant limitations in the AHI's metric properties. It is a poor predictor of key clinical outcomes such as EDS, hypertension, health‐related quality of life, and treatment response. Consequently, the assumed dose–response relationship between the frequency of respiratory events and clinical impact appears to be flawed. Reliance on the AHI leads to an overestimation of OSA's clinical relevance (Pevernagie et al. 2020).

Scientific interest has meanwhile shifted from merely counting respiratory events to understanding the pathophysiologic exposures they induce, particularly their systemic effects. It is now presumed that biomarkers of exposure may offer improved prediction of OSA severity. Growing evidence highlights the central role of intermittent hypoxia in driving cardiovascular comorbidities. Emerging biomarkers, such as hypoxic burden, may provide a more accurate assessment of the clinical impact of OSA (Stanczyk et al. 2025). However, clinical observations at the individual patient level reveal considerable variability and unpredictability also in the relationship between the degree of hypoxic exposure and symptom burden. Although markers of hypoxia may outperform the AHI in some respects, their overall predictive power is likely to remain limited.

The frequently observed mismatch between OSA severity, as assessed by pathophysiological markers, and a patient's symptom profile suggests the involvement of additional, less apparent factors. These factors may elude detection through conventional physiological recordings, as they could stem from an individual's genomic constitution or from mechanisms that are not captured in routine clinical assessments of sleep. Recent evidence suggests that certain vulnerability factors modulate the association between performance on cognitive testing and the severity of OSA (Marchi et al. 2024). Such underlying influences may be relevant as, ultimately, they could determine a person's susceptibility vs. resilience to the harmful systemic effects of OSA. Nonetheless, research in this area remains in its early stages.

In light of these insights, it may be time to rethink the disease model of OSA. At its core, OSA is driven by repetitive physiological strain caused by respiratory events and their adverse systemic effects. This chronic, night‐by‐night strain gradually inflicts damage on the body. However, the extent and progression of end‐organ injury are shaped not only by the magnitude of the strain but also by the intrinsic resilience of the affected organs. It is this dynamic interplay between chronic stress and individual resilience that ultimately determines the clinical manifestations of OSA in the course of time.

**FIGURE 2:**
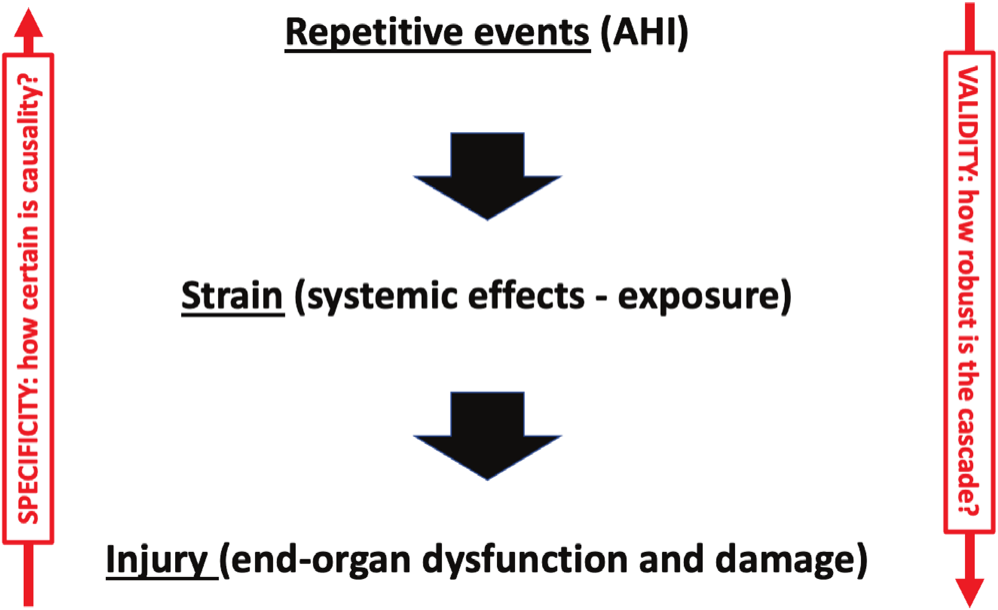
Obstructive sleep apnoea—A repetitive strain injury model.

A repetitive strain injury (RSI) model of OSA, illustrated in Figure 2, conceptualises the disease mechanism as a cascade. The process begins with the burden of repetitive respiratory events—a starting point that, on its own, has proven to be a poor predictor of clinical outcomes. The next stage in the cascade is the systemic strain, representing the cumulative exposure to the physiological consequences of these events. The final stage is injury, reflected in the clinical signs and symptoms associated with OSA.

When applying this model to individual patients, two key uncertainties arise. First, in the ‘downward’ direction: how robust and consistent is the pathogenic cascade from respiratory events over pathophysiological exposure to systemic injury? What is the validity of this RSI model? Second, in the ‘upward’ direction: to what extent can observed symptoms and clinical signs be confidently attributed to OSA, given their often non‐specific nature and potential overlap with other conditions?

Currently, these uncertainties pose significant challenges in clinical practice. While the ‘repetitive strain’ component can be measured with reasonable accuracy using existing technologies, the prediction of downstream ‘injury’ remains imprecise. To enhance clinical decision‐making, additional biomarkers with validated prognostic value will be required. Similarly, a satisfying therapeutic response to CPAP or alternative treatments cannot be assumed unless it is clear that the symptoms stem from OSA. If the manifestations are unrelated, treatment may yield disappointing results. Therefore, there is also a need for the development and validation of markers that add specificity to the causal link between OSA and its clinical manifestations. Such markers would significantly improve the ability to predict treatment outcomes and personalise care.

### 4.6 Paediatric Sleep Disorders

Paediatric sleep medicine faces several challenges. Sleep disorders in children, including insomnia, parasomnias, and sleep‐disordered breathing, are frequently underdiagnosed or misattributed to behavioural issues. This is partly due to limited paediatric sleep training among primary care providers, resulting in missed opportunities for early intervention. Adding to the problem is the scarcity of specialised paediatric sleep centres, and, in existing centres, the limited access to diagnostic and therapeutic services. PSG and CBT‐I are not widely available, with long waiting times and few public health options. In addition, multidisciplinary care models remain rare, leading to fragmented and inefficient service delivery (Reynolds et al. 2023).

These challenges collectively delay accurate diagnoses, allow sleep problems to persist or worsen, and contribute to comorbid conditions. They also increase the burden on healthcare providers, who must correct misinformation and rebuild trust with families.

The lack of trained professionals further impairs the implementation of evidence‐based treatments, making interventions less effective. Left untreated, paediatric sleep disorders can lead to increased healthcare utilisation, hinder cognitive and emotional development, impair academic performance, and contribute to long‐term societal costs.

Furthermore, pharmacotherapy in paediatric sleep disorders has often followed extrapolation from adult data or anecdotal practice. Many existing studies are short in duration, underpowered, and focused on symptom relief rather than developmental outcomes.

Paediatric sleep medicine requires prioritisation at the public health level through improved medical training, equitable access to resources, and integrated care models to address systemic challenges efficiently. Finally, emerging technologies like AI hold promise for improving diagnostic accuracy, but their application in paediatric sleep medicine must be carefully validated to ensure safety and effectiveness (Schlarb et al. 2025).

## 5 The Future Scope of Sleep Medicine

While sleep medicine is a rapidly evolving field, it struggles with finding a balance between huge potential on the one hand and intrinsic uncertainty on the other. Challenges especially pertain to further exploration of the areas of uncertainty, technological innovation, organisation of the field, as well as a transition towards precision medicine.

### 5.1 Resolution of Areas of Uncertainty

Some sleep disorders, for example, parasomnias, remain poorly understood due to the challenges in diagnosing them—often only by exclusion—and the inconsistent clinical experiences reported globally (Hrozanova et al. 2019). Advancing our understanding in this area will require increased research funding, enhanced cross‐disciplinary and interinstitutional collaboration, as well as a stronger emphasis from the medical community on recognising sleep as a fundamental component of overall health.

In OSA and other domains of sleep medicine, the main focus of investigation has been on male patients. This male‐predominant bias has led to a stark underrepresentation of females and children in epidemiologic studies and clinical sleep medicine reports (Hasuneh et al. 2024; Magnusdottir and Hill 2024; Moscucci et al. 2025). It has become evident that these populations often present with distinct phenotypic features compared to adult males. For instance, while men with OSA tend to be sleepy and typically exhibit loud snoring and witnessed apneas, women are more likely to report symptoms such as insomnia, fatigue, morning headaches, and mood disturbances, which may lead to misdiagnosis or underdiagnosis (Jordan et al. 2014; Pavlova and Sheikh 2011). Similarly, children with OSA may exhibit behavioural problems, hyperactivity, or learning difficulties rather than classic respiratory symptoms (Marcus et al. 2012). Obviously, these knowledge gaps must be addressed to improve diagnostic accuracy and to develop personal therapeutic approaches for women and children.

Last but not least, there are gaps in knowledge regarding the diagnosis, management and clinical significance of sleep‐disordered breathing in subjects with intellectual disability (Riha et al. 2024) and during different physiological states, for example, ageing and extreme old age as well as menopause and pregnancy (Johns et al. 2022). Work in these areas is sparse, and a more consolidated effort would be required to manage clinical implications and to resolve many questions that remain unanswered to date.

### 5.2 Technological Innovation

As in other domains of healthcare, sleep medicine is situated in a metamorphic environment, driven by rapid advancements in technology that are reshaping how disorders are being diagnosed, monitored, and treated. Several areas of interest are under development and tend to converge, that is, wearable technology, application of AI, remote monitoring, digital therapeutics, and advanced diagnostics. This evolution holds promise for enhancing accessibility, personalisation, and efficiency across the field. While several branches of academia and industry are involved worldwide, the Sleep Revolution project is worth mentioning in this context.

To address prevailing challenges in OSA, the Sleep Revolution project was funded in 2021 by the EU's Horizon 2020 research and innovation programme (Arnardottir et al. 2022; Sleep_Revolution 2021; Sleep_Revolution_EU 2021). The consortium consists of 39 partners from Europe and Australia, and combines multidisciplinary competencies and resources from academia, healthcare, and industry. The project aims to fundamentally change the clinical management of SDB by introducing a new diagnostic and digital management paradigm. It offers a comprehensive scope of diagnostic investigations, including a mobile application with a sleep diary and objective cognitive tests, a novel sleep questionnaire, a smart watch, and PSG recording in the home setting. A digital platform functions as a bridge between researchers, patients, and clinicians. Moreover, advanced telemedicine technology, novel machine learning algorithms for diagnosis, and a high degree of participatory patient involvement are combined in several clinical studies. The overall goal is to enable personalised treatment, enhance patient engagement, reduce diagnostic costs, and improve access to care. The initiative aims to establish new standardised guidelines in sleep medicine.

### 5.3 Organisation of Sleep Medicine as a Discipline

As described above, sleep medicine is founded on multidisciplinary interaction. Yet, interdisciplinary sleep healthcare remains inconsistent and fragmented. Many sleep practitioners focus routinely on a single aspect of sleep medicine, for example, CPAP treatment of OSA or CBT‐I (Edinger et al. 2021). The establishment of a unifying training pathway, as discussed above, for all practitioners working in the field would contribute substantially to interdisciplinary work, better understanding of the pathophysiology of most relevant sleep disorders, and more holistic care of individual patients (De Backer et al. 2011; Pevernagie et al. 2009; Strohl 2011). Formal education in sleep medicine should already start in undergraduate and continue in postgraduate healthcare training.

In settings where multidisciplinary sleep medicine is practised, integrated healthcare can be assured by implementing applicable standards. Sleep centre accreditation ensures the quality of diagnosis and treatment in sleep medicine. Originally introduced in Europe in 2006 for centres performing inpatient PSG and vigilance tests (Pevernagie and Steering Committee of European Sleep Research 2006), the standards have since been updated to reflect changes in clinical practice, including the widespread use of ambulatory diagnostics. In the recently revised European accreditation guideline, a tiered system was introduced (Hartley et al. 2024). Levels 1 and 2 sleep centres provide full laboratory‐based diagnostics, with level 1 typically being university‐affiliated and involved in teaching and research. Level 3 and 4 centres offer a mix of inpatient and ambulatory testing, with level 3 performing PSG and level 4 focusing on polygraphy, often for detecting OSA. Accreditation emphasises the importance of trained medical teams, proper equipment, patient care pathways, and adherence to national and European guidelines.

Finally, advocacy for sleep medicine should be extended to regulatory administrations at a national level. Healthcare authorities should be briefed about the adverse clinical and socio‐economic outcomes of untreated sleep disorders. Besides enhancing sleep medicine education among healthcare professionals across various medical disciplines, the establishment of sleep medicine as a discipline in its own right is the ultimate goal. Sleep medicine has become an authorised medical subspecialty in France and Germany. In many other European countries, sleep medicine is still off the grid in the national healthcare system. The ESRS fosters the accreditation of sleep medicine at a European level. An *ad hoc* taskforce has been appointed to approach the European Union of Medical Specialists (UEMS) and to accredit sleep medicine as a multidisciplinary joint committee (UEMS 2025). Being accredited by the UEMS will offer opportunities for official recognition of sleep medicine in other European countries and for international harmonisation of educational programmes.

### 5.4 Transition Towards Precision Medicine

The sleep medicine field is intrinsically complex. Diagnostic procedures are intensive and possibly already beyond their expiration date. The adoption of newer, more precise approaches to enhance both accuracy and accessibility is required. The demand for innovation necessitates not only technical development but also identification of novel markers that enhance diagnostic specificity and prognostic relevance.

The quest for suitable biomarkers is paramount in contemporary sleep medicine. A biomarker is defined as ‘a characteristic that is objectively measured and evaluated as an indicator of normal biological processes, pathogenic processes, or pharmacological responses to a therapeutic intervention’ (Biomarkers Definitions Working 2001; Califf 2018; Strimbu and Tavel 2010).

Hypocretin‐1 can be measured in the cerebrospinal fluid. It is a sensitive and specific indicator, as a level below 110 pg/mL has high accuracy for the diagnosis of narcolepsy type 1 in adults. By contrast, the AHI fails as a biomarker in corroborating causality of clinical manifestations and severity of SDB. However, the AHI is a sensitive indicator of therapeutic adequacy, as it will usually drop to normal values if treatment with CPAP or MRA is efficacious. Therefore, the AHI should not be discarded from the diagnostic framework but may be repurposed as an outcome variable. This approach may also refine the concepts of therapeutic outcomes.

**FIGURE 3:**
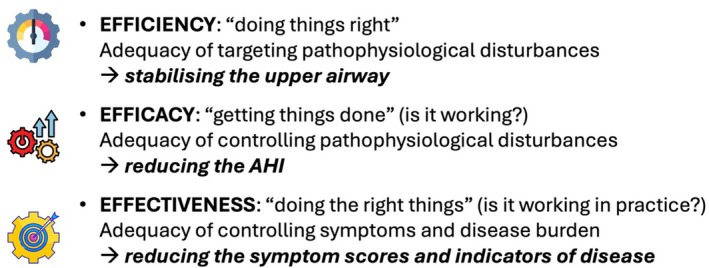
Different meanings of efficiency, efficacy and effectiveness.

Using pathophysiologic indices such as the AHI for the assessment of therapeutic effects enables the discrimination of efficacy versus effectiveness of treatment. The terms efficiency, efficacy, and effectiveness are often used interchangeably, but they have distinct meanings, especially in the context of research, healthcare, business, and policy analysis (Figure 3) (Patel 2021). In the clinical setting, ‘efficacy’ means that the treatment is successful at controlling the pathogenetic mechanism (e.g., normalising the AHI in OSA). ‘Effectiveness’ means that the treatment also results in adequate improvement of symptoms and signs (e.g., reducing sleepiness and high blood pressure in OSA).

Distinguishing between these aspects is crucial, as treatments may differ in their physiological impact while yielding similar clinical outcomes. For example, treatment with MRA often provides comparable symptomatic relief to CPAP, despite achieving a smaller reduction in the AHI (Ramar et al. 2015). Confusion arises when efficacy—defined as the degree of pathophysiological control—is inconsiderately equated with effectiveness, which pertains to clinical benefit experienced by the patient. In OSA, the concept of ‘mean disease alleviation’ implies that a partial decrease of the AHI under treatment can signify an overall reduction in disease burden (Budhiraja et al. 2025). However, this assumption holds only if clinical symptoms improve in parallel. If symptoms such as EDS persist despite adequate AHI control, the concept becomes ambiguous. Indeed, it is well established that OSA treatment may be efficacious in physiological terms but ineffective in alleviating symptoms of sleepiness (Bonsignore et al. 2021). This potential disconnect between efficacy and effectiveness is particularly relevant for non‐CPAP therapies aimed at specific endotypic traits in OSA. Whether such treatments, while being efficacious, also produce meaningful symptomatic relief remains subject to further investigation (Stanczyk et al. 2025).

Specificity enhancing biomarkers will have to be found in many areas of sleep medicine, for example, sleep deprivation, sleep disturbances in psychiatric disorders, SDB and chronic insomnia. However, relevant biomarkers for sleep disorders are hard to find (Borker and Strohl 2025). Taking OSA as an example, systematic reviews studying several candidate protein and genomic markers have found inconsistent findings and have not resulted in either sensitive or specific actionable markers to predict or intercede on comorbidity development.

In the future, precision medicine may be envisaged as a new paradigm to overcome the limitations of the current diagnostic workup. Precision medicine is defined as ‘treatments targeted to the needs of individual patients on the basis of genetic, biomarker, phenotypic or psychosocial characteristics that distinguish a given patient from other patients with similar clinical presentations’, the main objective being to ‘improve clinical outcomes for individual patients while minimizing unnecessary side effects for those less likely to respond to a given treatment’ (Jameson and Longo 2015). The rationale for embracing precision medicine is the observation that chronic diseases are ‘complex’ and ‘heterogeneous’. In this setting, ‘complex’ means that they have several components with nonlinear dynamic interactions, whereas ‘heterogeneous’ indicates that not all of these components are present in all patients or, in a given patient, at all timepoints. Obviously, many sleep disorders meet this definition of chronicity. While precision medicine is emerging on the horizon, the way to this destination will be long and uncertain.

In summary, sleep medicine stands at a crossroads. Its future will depend on redesigning the mission and vision of the field, remedying prevailing misconceptions, confronting structural challenges, embracing innovation, and validating its crucial role in overall health.

## 6 Conclusion

The name ‘sleep medicine’ refers to a horizontally structured clinical specialty built on an interdisciplinary approach to diagnosing and managing patients with diverse sleep disturbances. The medical significance of sleep disorders has been recognised since the 19th century, and recent years have brought substantial advances in understanding the underlying disease mechanisms.

Core diagnostic methods include detailed clinical history and physiological sleep recordings. While these tools generally support accurate diagnosis and effective treatment planning, the absence of reliable biomarkers for disease identification or prognosis continues to limit clinical precision.

Despite these challenges, sleep medicine has evolved into a well‐structured discipline, with official accreditation in several European countries. Yet, its foundation in medical education remains critical. The continued development of clinical practice guidelines and international harmonisation efforts are paving the way for sleep medicine to become an established and enduring component of modern healthcare.

## Author Contributions

**Dirk A. Pevernagie:** conceptualization, writing – original draft, methodology, writing – review and editing, supervision. **Erna Sif Arnardottir:** conceptualization, writing – original draft, methodology, writing – review and editing. **Oliviero Bruni:** conceptualization, writing – original draft, methodology, writing – review and editing. **Sarah Hartley:** conceptualization, writing – original draft, methodology, writing – review and editing. **Gert Jan Lammers:** conceptualization, writing – original draft, methodology, writing – review and editing. **Tiina Paunio:** conceptualization, writing – original draft, methodology, writing – review and editing. **Dieter Riemann:** conceptualization, writing – original draft, methodology, writing – review and editing. **Renata L. Riha:** conceptualization, writing – original draft, methodology, writing – review and editing, supervision.

## Conflicts of Interest

The authors declare no conflicts of interest.

## Data Availability

Data sharing is not applicable to this article as no new data were created or analyzed in this study.
